# Subtypes of anterior circulation large artery occlusions with acute brain ischemic stroke

**DOI:** 10.1038/s41598-020-60399-3

**Published:** 2020-02-26

**Authors:** Kun Zhang, Tong Li, Jing Tian, Peifang Li, Baosheng Fu, Xiaoli Yang, Luji Liu, Yanying Zhao, Honglin Lu, Pandi Zhao, Kailin Bu, Zhongzhong Li, Si Yuan, Qisong Wang, Yingzhen Zhang, Li Guo, Xiaoyun Liu

**Affiliations:** 1 Department of Neurology, The Second Hospital of Hebei Medical University, 215 West Heping Road, Shijiazhuang, Hebei 050000 China; 2 Neuroscience Research Center, Medicine and Health Institute, Hebei Medical University, 361 East Zhongshan Road, Shijiazhuang, Hebei 050000 China; 3 Hebei Medical University, 361 Zhongshan East Road, Shijiazhuang, Hebei 050000 China

**Keywords:** Stroke, Risk factors

## Abstract

Anterior circulation large artery occlusion (AC-LAO) related acute ischemic stroke (AIS) is particularly common in clinics in China. We retrospectively analyzed 787 consecutively hospitalized AIS patients with AC-LAO in Hebei Province, China. AC-LAO was defined as a complete occlusion of at least one intracranial internal carotid artery (ICA) or middle cerebral artery (MCA) based on computed tomography or magnetic resonance angiography. Among eight subtypes of AC-LAO, unilateral MCA occlusion is the most common one (49.8%, n = 392), while bilateral ICA/unilateral MCA occlusion is the least (0.3%, n = 2). Compared with unilateral MCA and unilateral ICA occlusion, patients with tandem ICA/MCA and bilateral ICA/MCA occlusion had poor outcomes after suffering AIS. Age (OR 1.022; 95%CI, 1.007 to 1.036) was an independent risk factor for single artery progressed to multiple artery occlusion, while ApoA1 (OR 0.453; 95% CI, 0.235 to 0.953) was a protective factor. Patients with unilateral MCA occlusion were prone to artery-to-artery embolism infarction subtype, unilateral ICA occlusion group were the most vulnerable to hypoperfusion/impaired emboli clearance subtype. Our results suggested various AC-LAO subtypes have different clinical characteristics and prognosis and were prone to different subtypes of infarction. Customized preventive measures based on AC-LAO subtypes may be more targeted preventions of stroke recurrences for AIS patients and could improve their prognoses.

## Introduction

Anterior circulation large artery occlusion (AC-LAO) is the most common cause of ischemic strokes^1,2^, especially in Chinese population^3^. For the treatment of those patients, thrombolytic therapy has a time window, and endovascular intervention treatment requires advanced surgical equipment and intensive care. Due to the large differences in medical conditions and levels across China, endovascular intervention treatment is available only in major stroke centers in cities, which means it cannot be applied to all patients with acute infarction, especially in rural areas^4–6^. Previous study showed intravenous thrombolysis and endovascular intervention treatments may not be effective in most patients with high clot burden LAO, and the resulting emboli may cause a worse prognosis^7^. Thus, there is still no treatment much more effective for patients with LAO suffering acute ischemic stroke (AIS) currently. By reviewing the literature, we found that most studies focusing on single unilateral middle cerebral artery (MCA), unilateral internal carotid artery (ICA), or tandem ICA/MCA occlusion, showing that a thrombus in the more proximal intracranial vasculature were more likely to have a poor outcome^8–10^. However, in clinical work we found that there are some other types of AC-LAO, such as unilateral and/or bilateral MCA combining unilateral and/or bilateral ICA. Whether different infarction subtypes are related with different types of occlusions or whether the prognosis worsens with the degree of artery occlusion have not been studied in depth until now. Therefore, we believe that it is necessary to conduct clinical characteristic and prognosis studies on different types of AC-LAO with AIS. Detailed classification analysis is conducive to accurate treatment and prognostic evaluation. Thus, our aim of this study was to investigate the clinical characteristics of different subtypes of AC-LAO with acute brain ischemic stroke, and to further analyze the risk factors and prognosis of different subtypes of AC-LAO related with AIS.

## Patients and Methods

**Figure 1:**
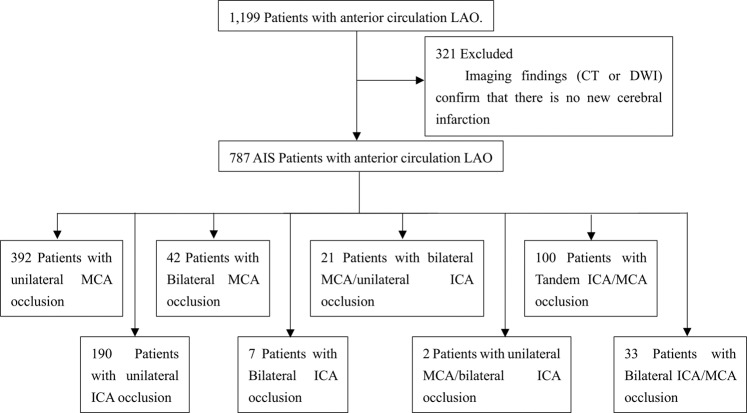
Flow-chart of search and screening process.

We retrospectively evaluated 787 consecutively hospitalized AIS patients with AC-LAO in The Second Hospital of Hebei Medical University, in North China, between June 1st, 2016 and April 30th, 2018. AC-LAO was defined as the presence of complete occlusion of at least one unilateral intracranial ICA or MCA based on CTA or MRA, and the criterion of AIS was new cerebral infarction confirmed with CT and/or DWI results^11^. ICA occlusion refers to the complete occlusion of the C1-C7 segment of the internal carotid artery shown in MRA or CTA. Flow-chart of search and screening process is shown in Fig. 1. After approval from the research ethics committee, the data were collected from hospital internal database for analysis.

**Figure 2:**
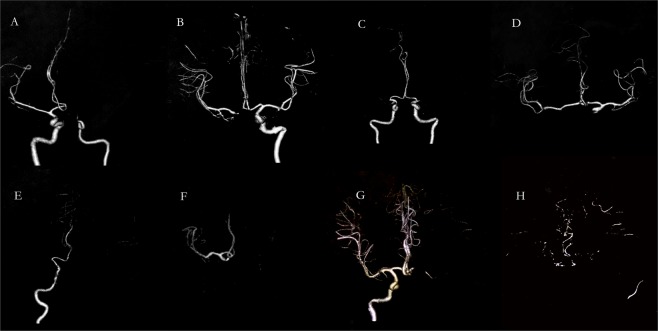
Eight anterior circulation LAO types: (**A**) unilateral MCA, (**B**) unilateral ICA, (**C**) bilateral MCA, (**D**) bilateral ICA, **(E**) bilateral MCA/unilateral ICA, (**F)** bilateral ICA/unilateral MCA, (**G**) tandem ICA/MCA, and (**H**) bilateral ICA/MCA.

According to which arteries were involved by occlusion all patients were classified into the following eight types: (1) unilateral MCA (n = 392), (2) unilateral ICA (n = 190), (3) bilateral MCA (n = 42), (4) bilateral ICA (n = 7), (5) bilateral MCA/unilateral ICA (n = 21), (6) bilateral ICA/unilateral MCA (n = 2), (7) tandem ICA/MCA (n = 100), and (8) bilateral ICA/MCA (n = 33) (Fig. 2), whose baseline characteristics are described in Table 1 by groups.

**Table 1:** Baseline characteristics of different artery occlusion groups.

Occlusive artery type	Unilateral MCA (n = 392)	Unilateral ICA (n = 190)	Bilateral MCA (n = 42)	Bilateral ICA (n = 7)	Bilateral MCA/unilateral ICA (n = 21)	Tandem ICA/MCA (n = 100)	Bilateral ICA/MCA (n = 33)	*P* value
Age	58.4 ± 12.6	59.3 ± 11.3	55.6 ± 12.2	66.9 ± 10.5	62.4 ± 11.6	64.2 ± 10.2	63.1 ± 10.0	<0.001***
Gender (male)	64.8% (n = 254)	76.8% (n = 146)	47.60% (n = 20)	85.7% (n = 6)	61.9% (n = 13)	69.0% (n = 69)	69.70% (n = 23)	0.006**
Length of stay (days)	14.0 ± 8.3	13.9 ± 7.2	11.5 ± 4.4	11.1 ± 7.1	14.4 ± 5.2	14.2 ± 14.6	11.9 ± 5.6	0.118
Smoking	34.3% (n = 134)	47.4% (n = 90)	16.7% (n = 7)	42.9% (n = 3)	19.0% (n = 4)	34.0% (n = 34)	30.3% (n = 10)	0.002**
Drinking	29.9% (n = 117)	35.8% (n = 68)	16.7% (n = 7)	28.6% (n = 2)	19.0% (n = 4)	29.0% (n = 29)	27.3% (n = 33)	0.242
BMI	25.3 ± 3.8	25.7 ± 3.9	25.8 ± 2.5	26.6 ± 3.2	25.3 ± 3.2	24.8 ± 4.2	24.2 ± 5.7	0.191
Hypertension	67.3% (n = 264)	61.6% (n = 117)	66.7% (n = 28)	71.4% (n = 5)	66.7% (n = 14)	69.0% (n = 69)	66.7% (n = 22)	0.870
Diabetes	22.4% (n = 88)	26.8% (n = 51)	35.7% (n = 15)	0.0% (n = 0)	28.6% (n = 6)	27.0% (n = 27)	33.3% (n = 11)	0.230
Heart disease	11.3% (n = 44)	18.4% (n = 35)	16.7% (n = 7)	14.3% (n = 1)	4.8% (n = 1)	17.0% (n = 17)	18.2% (n = 6)	0.215
Hyperlipidemia	29.1% (n = 114)	34.2% (n = 65)	21.4% (n = 9)	42.9% (n = 3)	33.3% (n = 7)	31.3% (n = 31)	48.5% (n = 16)	0.200
LDL	2.8 ± 0.9	2.8 ± 1.0	2.8 ± 0.9	2.9 ± 0.6	3.0 ± 1.0	2.8 ± 1.0	3.0 ± 0.8	0.687
HHCY	46.0% (n = 180)	44.2% (n = 84)	50.0% (n = 21)	42.9% (n = 3)	38.1% (n = 8)	48.0% (n = 48)	33.3% (n = 11)	0.782
ApoB	1.0 ± 0.3	1.0 ± 0.4	0.9 ± 0.2	0.9 ± 0.3	1.0 ± 0.3	0.9 ± 0.3	1.0 ± 0.3	0.195
ApoA1	1.2 ± 0.2	1.1 ± 0.2	1.1 ± 0.2	1.1 ± 0.4	1.1 ± 0.2	1.1 ± 0.2	1.1 ± 0.2	0.207
ApoB/ApoA1	0.9 ± 0.3	0.9 ± 0.3	0.8 ± 0.3	0.9 ± 0.4	0.9 ± 0.3	0.8 ± 0.3	1.0 ± 0.4	0.507
History of ischemic stroke	34.9% (n = 137)	24.3% (n = 46)	53.7% (n = 22)	71.4% (n = 5)	47.6% (n = 10)	41.0% (n = 41)	39.4% (n = 13)	0.001**
Complication	18.9% (n = 74)	14.7% (n = 28)	26.2% (n = 11)	28.6% (n = 2)	28.6% (n = 6)	29.0% (n = 29)	36.4% (n = 12)	0.014*
Single or multiple infarction (multiple)	80.9% (n = 317)	86.8% (n = 165)	71.4% (n = 30)	100.0% (n = 7)	100.0% (n = 21)	82.0% (n = 82)	66.7% (n = 22)	0.006**
ASPECTS	5.5 ± 2.9	6.2 ± 2.6	6.5 ± 2.8	7.4 ± 1.6	4.8 ± 2.6	4.2 ± 3.3	4.8 ± 3.4	<0.001***
NIHSS	7.3 ± 7.2	6.8 ± 6.6	7.1 ± 6.9	6.4 ± 12.7	7.4 ± 7.8	10.8 ± 8.1	10.7 ± 7.6	<0.001***
mRS	2.8 ± 1.5	2.7 ± 1.5	3.1 ± 1.3	1.7 ± 1.8	2.6 ± 1.4	3.4 ± 1.2	3.7 ± 1.4	<0.001***

MCA: Middle cerebral artery; ICA: Internal carotid artery; BMI: Body mass index; LDL: Low-density lipoprotein; HHCY: hyperhomocysteinemia; AF: Atrial fibrillation; ASPECTS: Alberta stroke program early CT score; NIHSS: National Institutes of Health Stroke Scale; mRS: Modified Rankin Scale. *p < 0.05, **p < 0.01, ***p < 0.001.

In order to explore potential correlations between AC-LAO subtypes and infarction subtypes, according to Chinese ischemic stroke sub-classification (CISS) system, 787 LAO patients with acute cerebral infarction were categorized into: (a) parent artery (plaque or thrombus) occluding a penetrating artery, (b) artery-to-artery embolism, (c) hypoperfusion/impaired emboli clearance, and (d) multiple mechanisms. A neurologist proficient in CISS classification performed CISS classifications for all patients based on their clinical symptoms, previous medical history, type of infarction of the imaging findings, and related signs and auxiliary examinations. These subtypes were defined according to the underlying mechanisms with which ischemic stroke is caused by large-artery atherosclerosis^12^.

The aim in this study is to retrospectively analyze the subtypes of AC- LAO in detail and find out if a patient’s existing subtype is related to his infarction type and the different prognosis of patients with different AC-LAO subtype. Informed consent was obtained from all patients or their relatives. The study protocol was approved by the local ethics committee (Research Ethics Committee of the second hospital of Hebei Medical University, approval No. 2018-P043), and all our methods and procedures were conducted in close accordance with all guidelines and/or regulations that apply.

### Statistical analysis

Statistical analysis was performed using the SPSS statistics software (version 25.0, IBM, Armonk, NY, USA). Quantitative variables were expressed as means (SD) in the case of normal distribution, or medians (interquartile range) otherwise. Categorical variables were expressed as percentages (numbers). Baseline characteristics of all patients were described according to the LAO types. Statistical significance of intergroup differences was assessed by Pearson χ^2^ test for categorical variables, and by Mann-Whitney-U test or Kruskal-Wallis test for quantitative variables, as appropriate. According to the statistical differences of the variables across groups, clinical experience, and previous literature results, certain variables were selected for regression analysis. Binary logistic regression was used to estimate the impact, in terms of odds ratios, of possible determinants of LAO severity and poor outcomes in LAO patients with acute infarction. Chi square hypothesis testing was performed to analyze the correlation between AC-LAO and infarction subtypes, and the intergroup difference was assessed by adjusted standardized residuals in post hoc testing. We considered that the difference between the observed frequency and the expected frequency had statistical significance if the absolute value of adjusted standardized residual was larger than 2^13^.

### Ethical standards

The study protocol was approved by the local ethics committee (Research Ethics Committee of the second hospital of Hebei Medical University, approval No. 2018-P043). Informed consent was obtained from all patients or their relatives.

## Results

Baseline characteristics of different artery occlusion groups are described in Table 1. Unilateral MCA occlusion (49.8%, n = 392) was the most common type of AC-LAO, followed by unilateral ICA (24.1%, n = 190), tandem ICA/MCA (12.7%, n = 100), bilateral MCA (5.3%, n = 42), bilateral ICA/MCA (4.2%, n = 33), bilateral MCA/unilateral ICA (2.7%, n = 21), bilateral ICA (0.9%, n = 7), and bilateral ICA/unilateral MCA (0.3%, n = 2). Since only two patients were in the bilateral ICA/unilateral MCA group, this group was excluded for statistical analysis. Both patients with bilateral ICA/unilateral MCA occlusion were male, 47 and 61 years old, respectively. They had history of ischemic stroke, and the National Institutes of Health Stroke Scale (NIHSS) scores of 2 and 10 and modified Rankin Scale (mRS) scores of 1 and 4, respectively. Of these two patients, the infarction in one was caused by parent artery occluding a penetrating artery while the other by artery-to-artery embolism.

Differences were observed in age (*p* < 0.001), gender (*p* = 0.006), smoking habit (*p* = 0.002), history of ischemic stroke (*p* < 0.001), complication(*p* = 0.014), single or multiple infarction (*p* = 0.006), Alberta stroke program early CT score (ASPECTS) (*p* < 0.001), NIHSS score (*p* < 0.001) and mRS score (*p* < 0.001) among groups. The number of AIS patients with a history of ischemic stroke and complication was the lowest in unilateral ICA occlusion group. Patients of bilateral ICA occlusion group had the highest average ages (66.9 ± 10.5), while bilateral MCA occlusion group the lowest (55.6 ± 12.2). Bilateral ICA occlusion group had the highest male ratio (85.7%), while bilateral MCA occlusion group had the lowest male ratio again (47.6%). Patients with smoking habits were the most common in unilateral ICA occlusion group (47.4%), but least common in bilateral MCA group (16.7%). AIS patients with bilateral ICA occlusion had the highest incidence of ischemic stroke history (71.4%), and all of their imaging appeared as multiple infarction. Furthermore, they had the highest ASPECTS and lowest NIHSS and mRS scores, which may indicate that patients with bilateral ICA occlusion are prone to infarction, but the prognosis will be better than other AC-LAO subtypes.

**Figure 3:**
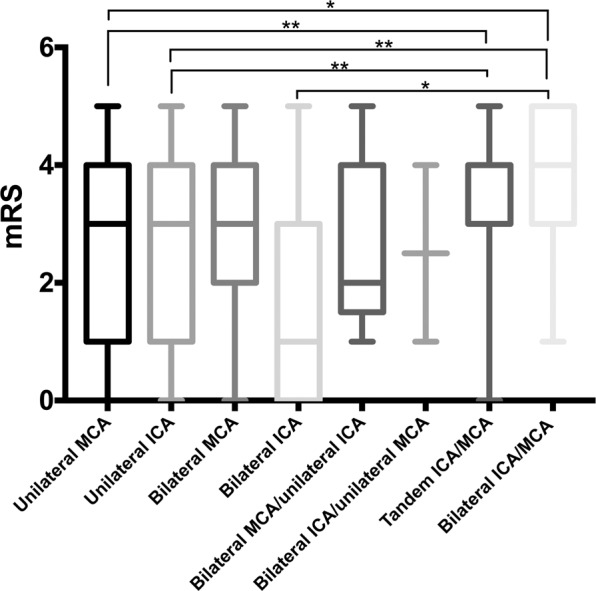
Multiple comparisons of prognosis of different AC-LAO subtypes. Compared with bilateral ICA/MCA group, unilateral MCA group, unilateral ICA group, and bilateral ICA group had better outcomes; tandem ICA/MCA group had poorer outcomes compared with unilateral MCA group and unilateral ICA group. The differences were statistically significant: *p < 0.05, **p < 0.01, ***p < 0.001.

The multiple comparisons about prognosis of different AC-LAO subtypes are shown in Fig. 3. Results showed that compared with unilateral MCA, AIS patients with tandem ICA/MCA(*p* = 0.0084) and bilateral ICA/MCA(*p* = 0.016) occlusion had worse outcomes. Similarly, AIS patients with unilateral ICA occlusion had better prognosis compared with tandem ICA/MCA(*p* = 0.001) and bilateral ICA/MCA(*p* = 0.004) groups, respectively. Also, compared with bilateral ICA group, bilateral ICA/MCA group had a better outcome (*p* = 0.0468), the difference was statistically significant.

Supplemental Table 1 summarizes the binary logistic-regression model for independent predictors of a poor outcome in acute infarction patients with AC-LAO. We deemed mRS greater than or equal to 4 a poor outcome. Long hospital stay (OR 1.04; 95% CI, 1.01 to 1.07), high NIHSS score (OR 1.37; 95% CI, 1.30 to 1.45), ASPECTS (OR 0.87; 95% CI, 0.81 to 0.93), and complications (OR 1.84; 95% CI, 1.11 to 3.05) were independent predictors of poor clinical outcomes. These results indicated that longer hospital stay and higher NIHSS were indicative of a higher likelihood for the patients with AC-LAO to suffer a poor outcome, and as ASPECTS increased, the patients were less likely to have a poor outcome. Besides, compared with patients without complications, those with complications were at a greater risk of a poor prognosis.

In order to investigate the risk factors of LAO severity, we then divided the eight LAO subtypes into single artery (including unilateral MCA and unilateral ICA) occlusion and multiple artery (other six subtypes) occlusion. The baseline characteristics are presented in Supplemental Table 2. Through chi-square or Mann-Whitney U tests, the two groups showed differences in the following aspects: age (*p* = 0.001), smoking habit (*p* = 0.017), ApoA1 level (*p* = 0.048), history of ischemic stroke (*p* < 0.001), ASPECTS score (*p* < 0.001), NIHSS score (*p* < 0.001), mRS score (*p* < 0.001), complication and infarction subtypes (*p* = 0.007). Further logistic regression analysis indicated age (odds ratio [OR] 1.022; 95% confidence interval [CI], 1.007 to 1.036) remained as an independent risk factor of multiple LAO, and ApoA1 (OR 0.453; 95% CI, 0.235 to 0.953) as an independent protect factor. Other relevant factors include NIHSS score (OR 1.030; 95% CI, 1.008 to 1.052) and infarction subtypes. These results suggested that patients with multiple LAO are more likely to suffer infarction caused by parent artery occluding penetrating artery, rather than by artery-to-artery embolism (OR 0.679; 95% CI, 0.475 to 0.971) and hypoperfusion/impaired emboli clearance (OR 0.473; 95% CI, 0.238 to 0.942) (shown in Table 2).

**Table 2:** Independent predictors of LAO severity in AIS patients.

	B value	Odds Ratio (95% CI)	*P* value
Age	0.021	1.022 (1.007–1.036)	0.009
ApoA1	−0.749	0.473 (0.235–0.953)	0.040
NIHSS	0.030	1.030 (1.008–1.052)	0.006
**Infarction subtypes**			
parent artery occluding penetrating artery			0.043
artery-to-artery embolism	−0.387	0.679 (0.475–0.971)	0.034
hypoperfusion/impaired emboli clearance	−0.748	0.473 (0.238–0.942)	0.033
multiple mechanisms	0.205	0.673 (0.473–3.191)	0.673

ASPECTS: Alberta stroke program early CT score; MCA: Middle cerebral artery; ICA: Internal carotid artery.

Furthermore, to investigate if LAO subtypes were related to infarction types, we analyzed the correlation between anterior circulation LAO type (bilateral ICA/unilateral MCA occlusion and bilateral ICA occlusion groups were excluded due to small sample sizes) and infarction subtype, and results suggested a weak correlation between anterior circulation LAO type and infarction subtype (χ^2^ = 25.4, Cramer’s V = 0.129, *p* = 0.005). Post hoc testing was performed to analyze intergroup differences (Table 3), and the results showed patients with unilateral MCA occlusion tended to experience infarction caused by artery-to-artery embolism (adjusted residuals = 2.8), rather than by parent artery occluding a penetrating artery (adjusted residuals = −2.2). The unilateral ICA occlusion group was susceptible to hypoperfusion/impaired emboli clearance-induced infarction (adjusted residuals = 3.1). Bilateral ICA/MCA occlusion was more likely to cause acute infarction via parent artery occluding a penetrating artery (adjusted residuals = 3.0) compared with artery-to-artery embolism (adjusted residuals = −2.0).

**Table 3:** Cross tabulation of occlusive artery types and infarction subtypes.

	parent artery occluding penetrating artery	artery-to-artery embolism	hypoperfusion/impaired emboli clearance
*Unilateral MCA*	184 (−2.2)	169 (2.8)	31 (−0.9)
*Unilateral ICA*	90 (−1)	68 (−0.8)	27 (3.1)
*Bilateral MCA*	24 (1.2)	13 (−0.8)	2 (−0.9)
*Bilateral MCA/unilateral ICA*	13 (0.9)	6 (−1)	2 (0.1)
*Tandem ICA/MCA*	57 (1.5)	34 (−0.9)	6 (−1)
*Bilateral ICA/MCA*	25 (3)	7 (−2)	0 (−1.8)

Note. Adjusted residuals appear in parentheses next to observed frequencies.

MCA: Middle cerebral artery; ICA: Internal carotid artery.

## Discussion

Intracranial LAO is the main cause of cerebral infarction. In China, the incidence of cerebrovascular disease due to intracranial LAO is higher than in Western countries^3^. Currently, most studies focused on acute brain ischemic stroke related with single-artery occlusion, such as MCA or ICA. In our study, we have cross-compared different LAO subtypes with AIS.

Japanese scholars have shown that the prognosis of AIS with LAO is gender-related: the prognosis of female patients after thrombolytic therapy is better^14^. Western studies have shown that such patients with low NIHSS and good collaterals achieve good long-term functional outcome^15^. In our results, high NIHSS was associated with multiple AC-LAO, and the patients with different AC-LAO subtypes showed different NIHSS, the average score of tandem ICA/MCA group is the highest and the bilateral ICA group is the lowest among eight subtypes. In addition, low ASPECTS and complication are also the independent risk factors for poor outcomes. As to why prognosis worsens as the ASPECTS decreases, according to previous studies, we considered it is associated with the absence of collaterals. Based on above, for AIS patients with LAO, we suggest emphasizing the evaluation of collaterals. On the other hand, our study shows that age was an independent risk factor for multiple AC-LAO, as single AC-LAO patients age, the possibility of complex multiple AC-LAO should be considered. Additionally, regression analysis shows ApoA1 was an independent protection factor for the progression of LAO. ApoA1 is the most important structural component of high-density lipoproteins (HDL) and plays a key role in the transport of cholesterol from peripheral tissues to the reverse cholesterol transport metabolism in the liver^16^. A large number of epidemiological studies have shown that plasma HDL levels are inversely associated with coronary artery disease^17^. The role of HDL in improving coronary artery disease is closely related to its main structural component ApoA1. According to a previously published study, the damaging mutation in ApoA1 increased risk of atherosclerosis^18^; even so, there is still controversy about the relationship between Apo and coronary heart disease^17^. From our results, we have reasons to believe that ApoA1 may be an important factor in preventing the progress of AC-LAO, and monitoring blood lipids, especially ApoA1, is very important in AIS patients with AC-LAO.

In addition to atherosclerosis, there are many other factors we should consider involved in LAO, such as immune factors and infections. Previous studies have shown that men account for a higher proportion of patients with intra- and extracranial atherosclerotic stenosis in China^19^. Our data indicate that except for bilateral MCA group (47.6%), all other groups contain more males than females, which agreed with previous studies. We know that female may be more prone to an inflammatory burden or to a vasculitis etiology of intracranial cerebrovascular conditions, because of hormonal factors and a difference in immune response. We analyzed 42 patients in the bilateral MCA group, 21 of whom were tested for serum autoantibodies and immunoglobulins. None of the results proved that the patients had autoimmune diseases. Three of them completed the virus series screening. Of them, a 44-year-old woman had serum CMV IgG, and HSV-1 IgG were significantly increased; RV IgG, CMV IgG, and HSV-1 IgG were significantly increased in a 39-year-old male; another 45-year-old male patient was diagnosed with treponema pallidum infection. Bilateral MCA occlusion in these three patients might have been related to viral or treponema pallidum infection. However, it is still difficult to determine the specific causes for this group of patients because not all patients have complete immunological test results. Other studies have observed that infections, such as syphilis^20^, tuberculosis^21^ and herpes virus^22^, can cause stenosis or occlusion of the intracranial arteries, increasing the risk of stroke in patients. It is suggested that in future clinical work, we can ask patients’ medical histories in more detail and screen atypical patients (young people without atherosclerotic risk factors with ischemic brain stroke) with unclear etiology. On the other hand, moyamoya disease should also be considered for multiple intracranial vascular occlusions. This disease is more common in Asian populations. Patients are younger and women experienced a higher incidence. Four of our patients were diagnosed with moyamoya disease before admission, three men and one woman, with an average age of 45.25 ± 13.25, two of whom had bilateral MCA occlusion and the other two had unilateral ICA occlusion. With the exception of a 47-year-old male (the mRS is four), the prognosis was good in three others. Considering the imaging results of CTA/MRA and clinical data, we cannot rule out the possibility of a certain period of moyamoya disease in our case. The histological characteristics of moyamoya disease and moyamoya syndrome caused by atherosclerosis may be different, and the physiopathological mechanism (unclear) may also affect the prognosis. Therefore, clinicians should think about the possibility of moyamoya disease when treating patients with AC-LAO. After the imaging, examination of immunological indicators and genetics should be performed to improve the diagnosis rate of moyamoya disease.

Different AC-LAO subtypes have different clinical manifestations, stroke types, and outcomes. Correlation tests revealed a correlation between occluded arterial classification and infarction subtype, which may suggest that preventive intervention can be instituted according to the type of occlusion in the patient to effectively prevent stroke recurrence. Patients with unilateral ICA occlusion were more likely to have cerebral infarction caused by hypoperfusion/impaired emboli clearance. Considering that these patients had integral bilateral MCA according to our classification, we speculate the possible reasons for hypoperfusion are as follows: 1. The primary collateral circulation, which involves the circle of Willis, cannot maintain sufficient perfusion, or the secondary collateral circulation is incompletely established^23,24^; 2. Thrombolysis from the unilateral ICA causes embolism after reaching bilateral MCA. For those patients, based on our clinical experience, moderately high levels of hypertension can be recommended to ensure intracranial perfusion, thereby preventing recurrence of cerebral infarction^25^. Patients with unilateral MCA occlusions were more likely to experience infarction due to artery-to-artery embolism, whereas patients with bilateral ICA/ MCA occlusion were more likely to experience infarction due to parent artery occluding a penetrating artery. We consider that it may be related to the mechanism of formation of atherosclerosis^26^. When early plaque formation causes occlusion of a susceptible artery, plaques are prone to rupture, leading to artery-to-artery embolism. As the situation worsens, bilateral ICA and bilateral MCA develop complete occlusion, and then continued atherosclerosis tends to affect the penetrating arteries. In such cases, smoke-like neovascularization may compensate for the blockage.

Our results suggested that various subtypes of AC-LAO have different susceptibility to different subtypes of infarction and outcomes, and further indicated that age promoted the progression of AC-LAO from single- to multiple-vessel occlusion, while ApoA1 suppressed this progression simultaneously. Therefore, we recommend monitoring and further looking for measures that can interfere with ApoA1 levels in LAO patients. Based on this situation, in addition to long-term intake of statins to lower blood lipids and stabilize plaques, lipid spectrum include Apoa1 levels should also be monitored for its effect for LAO severity according to our results. Depending on the subtype of AC-LAO which is related with the subtype of infarction, targeted preventive measures should be considered, which may effectively improve the prognosis of patients with LAO and reduce infarction recurrence.

In terms of study limitations, we included all classifications of anterior circulation large artery occlusion in order to conduct a complete analysis; however, because certain groups were too small in size to be entered into the statistical model, incomplete analysis may have resulted. Therefore, part of conclusions of this study needs to further verification with a larger sample size. Furthermore, pseudo-occlusion unavoidably appeared in few cases in this study, due to AC-LAO diagnosis based on CTA and MRA results (Of our patients with isolated ICA occlusion − 190 unilateral ICA occlusions and 7 bilateral ICA occlusions - 24 were performed DSA, and only a 60-year-old male was confirmed to be pseudo-occlusive. Since not all patients performed DSA and the number is small, we did not classify them as a group.). In addition, this was a cross-sectional, retrospective study of selected case data. We are following up with these patients, obtaining vascular imaging data and evaluating the level of collateral circulation, and striving to analyze patients with large artery occlusion for the purpose of identifying relevant factors to improve the long-term prognosis of such patients.

## Conclusion

In our study, we achieved a comprehensive description of the AC-LAO into different subtypes, and cross-compared the clinical features and prognosis of AC-LAO with AIS. Our study emphasized the division and understanding of different subtypes. Our results suggested besides high NIHSS, complication, long hospital stay, and low ASPECTS were associated with poor outcomes, different LAO subtypes had different infarction subtypes and outcomes, which may involve many factors including age, gender, smoking habit, and collaterals. In our future work, we will look deep into the mechanism of AC-LAO related AIS.

## Supplementary information

Supplementary information

## Data Availability

The data sets generated and analyzed during the current study are available on appropriate request made to the corresponding author.
