# Supplementary material for: Subtypes of anterior circulation large artery occlusions with acute brain ischemic stroke
Source: Sci Rep. 2020 Feb 26;10:3442. doi: 10.1038/s41598-020-60399-3 (PMC7044197; doi:10.1038/s41598-020-60399-3)
Supplement: Supplementary file 1 — Supplementary information [file 41598_2020_60399_MOESM1_ESM.pdf]

Title:

Subtypes of anterior circulation large artery occlusions with acute brain ischemic stroke

Authors:

Kun Zhang [1], Tong Li [1], Jing Tian [1], Peifang Li [1], Baosheng Fu [1], Xiaoli Yang [1], Luji Liu [1], Yanying Zhao [1], Honglin Lu [1], Pandi Zhao [1], Kailin Bu [1], Zhongzhong Li [1], Si Yuan [1], Qisong Wang [1], Yingzhen Zhang [3], Li Guo [1], Xiaoyun Liu [1, 2*]

[1] Department of Neurology, The Second Hospital of Hebei Medical University, 215 West Heping Road, Shijiazhuang, Hebei 050000, China

[2] Neuroscience Research Center, Medicine and Health Institute, Hebei Medical University, 361 East Zhongshan Road, Shijiazhuang, Hebei 050000, China

[3] Hebei Medical University, 361 Zhongshan East Road, Shijiazhuang, Hebei 050000, China

* Correspondence should be addressed to Xiaoyun Liu, MD, PhD, E-mail: audrey-l@163.com, Tel: 13191887318

Supplementary Table 1. Independent predictors of poor outcome of patients with large artery occlusion after acute infarction

	**B value**	**Odds Ratio (95% CI)**	***P* value**
Length of stay	0.040	1.041(1.011-1.071)	0.008**
Smoking habit	-0.407	0.666(0.440-1.008)	0.054
ASPECTS	-0.138	0.871(0.812-0.9342)	<0.001***
Complication	0.609	1.838(1.108-3.050)	0.018*
NIHSS	0.317	1.374(1.302-1.449)	<0.001***

ASPECTS: Alberta Stroke Program Early CT Score; NIHSS: National Institutes of Health Stroke Scale; MCA: Middle cerebral artery; ICA: Internal carotid artery

Supplementary Table 2. Baseline characteristics according to LAO severity

**Occlusive artery type**	**Single LAO(n=582)**	**Multiple LAO(n=205)**	***P* value**
Age	58.7±12.2	62.0±11.2	0.001**
Gender (male)	68.7%(n=400)	64.9%(n=133)	0.485
Length of stay (days)	14.0±8.0	13.1±10.9	0.177
Smoking	38.6%(n=224)	29.3%(n=60)	0.017**
Drinking	31.8%(n=185)	25.4%(n=52)	0.082
BMI	25.4±3.8	25.0±4.1	0.378
Hypertension	65.5%(n=381)	67.8%(n=139)	0.543
Diabetes	23.9%(n=139)	28.8%(n=59)	0.165
Heart disease	13.6%(n=79)	15.6%(n=32)	0.482
Hyperlipidemia	30.8%(n=179)	32.4%(n=66)	0.672
LDL	2.8±0.9	2.8±0.9	0.607
HHCY	45.4%(n=264)	45.4%(n=93)	0.986
ApoB	1.0±0.3	0.9±0.3	0.070
ApoA1	1.2±0.2	1.1±0.2	0.048*
ApoB/ApoA1	0.9±0.3	0.9±0.3	0.647
History of ischemic stroke	31.5%(n=183)	44.6%(n=91)	0.001**
ASPECTS	5.8±2.8	5.0±3.2	0.007**
NIHSS	7.2±7.0	9.5±8.0	< 0.001***
mRS	2.8±1.5	3.2±1.4	< 0.001***
Complication	17.5%(n=102)	29.3%(n=60)	< 0.001***
Multiple infarction	82.8%(n=482)	79.5%(n=163)	0.290
Infarction subtypes			0.007**
parent artery occluding penetrating artery	47.1%(n=274)	59.3%(n=121)	
artery-to-artery embolism	40.7%(n=237)	31.9%(n=65)	
hypoperfusion/impaired emboli clearance	10.0%(n=58)	5.4%(n=11)	
multiple mechanisms	2.2%(n=13)	3.4%(n=7)	

MCA: Middle cerebral artery; ICA: Internal carotid artery; BMI: Body mass index; LDL: Low-density lipoprotein; HHCY: hyperhomocysteinemia; AF: Atrial fibrillation; ASPECTS: Alberta stroke program early CT score; NIHSS: National Institutes of Health Stroke Scale; mRS: Modified Rankin Scale. *p<0.05, **p<0.01, ***p<0.001
